# ChIP-exo and CRISPRi/a illuminate the role of Pdr1 and Yap1 in acetic acid tolerance in *Saccharomyces cerevisiae*

**DOI:** 10.1128/aem.01824-24

**Published:** 2025-03-04

**Authors:** Ibai Lenitz, Christoph Börlin, Luca Torello Pianale, Darshan Balachandran, Jens Nielsen, Florian David, Verena Siewers, Yvonne Nygård

**Affiliations:** 1 Department of Life Sciences, Chalmers University of Technology 684649 , Gothenburg, Sweden; 2 BioInnovation Institute 633706 , Copenhagen, Denmark; 3 VTT Technical Research Centre of Finland 3259 , Espoo, Finland; Michigan State University, East Lansing, Michigan, USA

**Keywords:** yeast, inhibitor stress, transcription factor, expression, stress responses

## Abstract

Budding yeast *Saccharomyces cerevisiae* has great potential as a host organism for various biorefinery applications. Nevertheless, the utilization of renewable plant biomass as feedstock for yeast in industrial applications remains a bottleneck, largely due to the presence of inhibitory substances such as acetic acid that are released in the biomass pretreatment processes. Exposure to acetic acid leads to different cellular stress mechanisms, several of which are directed by transcription factors. In this work, the role of the transcription factors Pdr1 and Yap1 in acetic acid tolerance was investigated using ChIP-exo and CRISPR interference/activation (CRISPRi/a). Pdr1 is the main regulator of the pleiotropic drug response, whereas Yap1 governs the oxidative stress response. CRISPRa targeting *YAP1* for overexpression conferred a higher specific growth rate of *S. cerevisiae*, whereas CRISPRi-based downregulation of *PDR1* proved to be beneficial for growth in medium containing acetic acid. ChIP-exo experiments showed increased binding of Pdr1 or Yap1 to their target promoters in the presence of acetic acid, and a large number of promoters were bound by either transcription factor. Promoters of genes involved in amino acid synthesis or encoding ABC transporters had the highest level of binding enrichment in the presence of acetic acid. The results highlight the potential for developing more acetic acid-tolerant yeast by altering the expression of transcription factor-encoding genes and demonstrate how expression can be fine-tuned by CRISPRi/a.

**IMPORTANCE:**

Biotechnological conversion of plant biomass into a variety of commodity chemicals and specialty molecules is an important step towards a bioeconomy. This study highlights the importance of two transcription factors, Pdr1 and Yap1, in the tolerance of *Saccharomyces cerevisiae* to acetic acid, a common inhibitor in bioprocesses using lignocellulosic biomass. CRISPR interference/activation and ChIP-exo were used to manipulate the expression and binding of these transcription factors in response to acetic acid stress. The study provides new insights into adaptation to acetic acid and suggests ways to improve yeast performance in industrial applications.

## INTRODUCTION

*Saccharomyces cerevisiae* is extensively used as a production host in various biotechnological processes, such as production of biofuels, fatty acids, amino acids, or pharmaceuticals (1). Still, the sensitivity of *S. cerevisiae* to environmental stresses, such as acetic acid, hampers its full potential. Acetic acid is a common by-product of sugar fermentation and a major inhibitor in industrial bioprocess substrates, such as lignocellulosic hydrolysates made from plant biomass (2). Moreover, in bioprocesses in which high concentrations of acetic acid are produced, product inhibition may hamper the growth of the host cells (3). Acetic acid causes detrimental effects on various cellular processes and challenges the cells’ pH homeostasis, leading to decreased viability and productivity (4, 5).

Understanding the molecular mechanisms underlying *S. cerevisiae*’s tolerance to acetic acid and other lignocellulosic inhibitors has been the focus of many research efforts. Several transcription factors (TFs) have been identified to play a key role in acid tolerance (6). Haa1, a major regulator of the yeast acid stress responses, is involved in the activation of many acetic acid stress response genes, and overexpression of *HAA1* has been shown to improve tolerance to weak acid stress (7, 8). Less is known about the role of the TFs Yap1 and Pdr1 in acetic acid tolerance. While Pdr1 acts as the principal regulator of pleiotropic drug responses to diverse cytotoxic compounds (9), Yap1 orchestrates the activation of genes associated with antioxidant defense, reactive oxygen species (ROS) scavenging, and redox homeostasis (10). Deletion of *PDR1* has been shown to enhance cell survival in the presence of acetic acid (11). On the contrary, Semchyshyn et al. (12) reported reduced growth of an acetic acid-treated *Δyap1* strain. This underscores the complex and multifaceted nature of acetic acid stress adaptation and emphasizes the need for further investigations to understand the roles of Pdr1 and Yap1 in this process. Both Yap1 and Pdr1 have previously been reported to induce the activation of stress response genes in the presence of HMF, another lignocellulose-derived inhibitor (13, 14), highlighting the potential in engineering *YAP1* and/or *PDR1* expression as a means towards more tolerant *S. cerevisiae* cell factories for lignocellulosic biomass utilization.

Acetic acid has been reported to cause oxidative stress (2). Under oxidative stress, ROS accumulate in the cells, causing oxidative damage to proteins, lipids, and DNA (2). *YAP1* (Yeast Activator Protein 1) encodes a bzip (basic region leucine zipper) TF that binds to specific DNA sequences, called Yap Response Elements (YREs) in the promoters of genes that Yap1 regulates (15). This binding activates the transcription of genes involved in antioxidant defense (i.e., *TRR1*, *TRX2*, *GSH1*, and *GLR1*), encoding genes involved in thioredoxin reduction, glutamylcysteine synthesis, or glutathione reduction, which help to restore the cellular redox balance (10, 16–18). The transcriptional activity of Yap1 is controlled by its subcellular localization (19). The N-terminal region of Yap1 contains a nuclear localization signal, which facilitates its transport into the nucleus upon oxidative stress, while the C-terminal region of Yap1 contains a nuclear export signal (19, 20). Proper folding is needed for Yap1 to bind to YREs of target gene promoters (21).

The stress response of yeast forms a very complex regulatory network, where many TFs are connected. Yap1 has been demonstrated to be part of the transcriptional network of Rpn4, a TF controlling the expression of proteasomal genes. Additionally, Yap1 has been demonstrated to regulate Pdr1 (22, 23). We have previously shown that the regulation of proteasomal genes is crucial for weak acid tolerance in *S. cerevisiae* (24). Rpn4 has been proposed to connect the regulation of the proteasome with the oxidative stress response governed by Yap1 and the PDR network steered by Pdr1 (22). The PDR network governs multidrug resistance in *S. cerevisiae* through 10 TFs that regulate the expression of more than 70 target genes (9). Pdr1 (Pleiotropic Drug Resistance 1) is a zinc-finger TF that has been reported to be the main regulator of tolerance towards different cytotoxic drugs (9). Pdr1 has been described to directly regulate around 50 genes, many of which encode different transporters or genes involved in plasma membrane composition (25). Pdr1 also regulates genes encoding two other TFs of the PDR network, namely, *PDR3* and *YRR1*, which are also autoregulated (26, 27). Pdr1 and Pdr3 are functional homologs and bind to the same DNA recognition site called the Pdr1/Pdr3 response element (PDRE) (28). Notably, Pdr1 and Pdr3 can act as both transcriptional activators and repressors and form homo- and heterodimers (29). These TFs have overlapping but not identical sets of target genes and may regulate their target genes differently (30, 31).

The overexpression or deletion of various target genes, including several TFs, has been successfully employed for enhancing inhibitor tolerance in yeast (6). Some more recent studies employed CRISPR interference (CRISPRi) or CRISPR activation (CRISPRa) to study and improve inhibitor tolerance (24, 32–35). The CRISPRi/a technology uses a catalytically inactive Cas9 protein (dCas9) coupled with a transcriptional inhibitor/activator to achieve a transient change in expression of the target gene (36). This method allows for gradual modulation of the expression level, which can translate into more optimal gene expression levels compared to gene knockouts or promoter replacements that are typical in cell factory construction (37). As gRNA targeting efficiency has been reported to be highly dependent on the location and properties of binding motifs, multiple gRNAs are commonly used to effectively achieve transcriptional modulation (38–40). While the possibility to alter the expression of networks of genes by altering the expression of TFs is appealing, the underlying target genes of specific TFs are often still unknown.

The identification of binding sites for specific TFs can be achieved through chromatin immunoprecipitation (ChIP) methods. ChIP methods are based on cross-linking the DNA and the DNA-bound proteins with a chemical agent, typically formaldehyde, upon which the DNA-protein complexes are immunoprecipitated by specific antibodies (41). In ChIP-seq, the protein-bound DNA (including TF target sites) is identified by Illumina sequencing. In ChIP-exo, a variant of ChIP-seq, a lambda exonuclease treatment is introduced to digest unbound DNA, which enhances resolution to near-single-nucleotide levels (42, 43). Several large-scale studies of TF binding in *S. cerevisiae* have been conducted using various ChIP methods. Lee et al. (44) and Harbison et al. (23) investigated the binding of 203 TFs under various growth conditions. Another study focused on 30 TFs involved in the DNA damage response (45). Furthermore, the binding of Fkh1 and Fkh2, two TFs coordinating the cell cycle progression, was determined in both logarithmic and stationary phases using ChIP-exo (46).

In this study, we investigate the roles of two TFs, Pdr1 and Yap1, in *S. cerevisiae*’s tolerance to acetic acid stress. Through ChIP-exo analysis, we identified target genes bound by these TFs in the presence of acetic acid. Subsequently, employing CRISPRi and CRISPRa, we modulated the expression of *PDR1* and *YAP1*. A set of strains expressing the CRISPRi/a components and eight different gRNAs targeting the promoters of *PDR1* or *YAP1* were characterized for growth in the presence of acetic acid. Our findings contribute to a deeper understanding of the genetics underlying *S. cerevisiae*’s acetic acid tolerance that can be used for engineering more tolerant yeast strains.

## MATERIALS AND METHODS

### Yeast strain, oligonucleotides, and culture conditions

*Saccharomyces cerevisiae* CEN.PK 113-5D (47) was used as the parental strain. For CRISPRi/a, a total of eight sgRNAs were chosen within 400 bps from the Transcription Start Site (TSS), with one sgRNA targeting a sequence between the TSS and the start codon of the gene (Table S9). Oligonucleotides encoding sgRNAs or serving as PCR primers (Tables S8 and S9) were ordered from Eurofins. The sgRNAs were designed using CRISPR-ERA (48) and CHOP CHOP (49). pRS416-based vectors (TetR-dCas9-Mxi1 or TetR-dCas9-VPR) with the *URA3* as selection marker (50) were used for expression of dCas9 and the sgRNAs. Assembly of the vector and the sgRNA-encoding DNA fragments was carried out as previously described (50), using the NEBuilder HiFi DNA Assembly Master Mix (New England Biolabs, USA).

YNB medium (1.7 g L^−1^ YNB [BD Difco], 0.79 g L^−1^ complete supplement mixture (-uracil) [Formedium], 5 g L^−1^ ammonium sulfate, 10 g L^−1^ succinic acid, and 6 g L^−1^ sodium hydroxide) at pH 4.5 was used for precultures of the CRISPRi/a strains. The precultures were inoculated from glycerol stocks and incubated in 96-well plates at 30°C, shaking at 220 rpm, for 48 h. To set the concentration of acetic acid to be used for strain characterization, the YNB medium was supplemented with acetic acid at 0, 80, 100, 120, and 140 mM. These experiments were conducted with the control strain, containing the control plasmid with no sgRNA, in two independent 96-well plates with six replicates per condition. A concentration of 120 mM acetic acid was chosen for the screens, as this caused slower growth rates, but still allowed the strains to grow (Fig. S1). The cultures were inoculated at an OD_600_ of 0.1 in 250 µL liquid medium and incubated for 96 h at 30°C. The screens were conducted in biological triplicates, using a growth profiler 960 device (Enzyscreen). Data on biomass formation of the CRISPRi/a strains (Table 1) were registered every 30 min.

**TABLE 1:** Overview of tolerance screenings with CRISPRi/a strains^*a*^

Strain	Transcription factor	Modulation	CRISPRi/a component
PDR1i-sgRNA1-8	Pdr1	CRISPRi (downregulation)	dCas9-Mxi1
PDR1a-sgRNA1-8	Pdr1	CRISPRa (upregulation)	dCas9-VPR
YAP1a-sgRNA1-8	Yap1	CRISPRa (upregulation)	dCas9-VPR

^*a*^ All screens were done in medium supplemented with 120 mM acetic acid at pH 4.5.

For ChIP-exo experiments, minimal medium containing 2% glucose, 14.4 g/L KH_2_PO_4_, 0.5 g/L MgSO_4_, 7.5 g/L (NH_4_)_2_SO_4_, 1 mL/L trace metal stock solution, and 1 mL/L vitamin stock solution was used (51). Cells were grown overnight in minimal media and then diluted to an OD_600_ of 0.1 in media containing 40 mM of acetic acid. After 8 h of incubation, cells were harvested by centrifugation and frozen in liquid nitrogen.

### ChIP-exo and data analysis

Chromatin immunoprecipitation, DNA extraction, and library preparation for the ChIP-exo experiment were carried out as described in Liu et al. (52). The bioinformatic pipeline used to analyze the sequencing results was described in Börlin et al. (53). Target genes were identified as genes that had a mean TF-binding count in the region from −1,000 bp upstream of the TSS to 1,000 bp downstream of the TSS that was more than 2.5 times higher than the background TF-binding count (of all genes). Gene Ontology (GO) analysis of TF target gene-binding enrichment was done by introducing the gene targets bound solely in the presence of acetic acid to the Gene Ontology Term Finder (yeastgenome.org) and using all genes of the *S. cerevisiae* genome as the reference. GO terms with a corrected *P* value inferior to 0.1 were considered to be enriched.

### Analysis of CRISPRi/a strains characterized for growth

The growth data from the tolerance screens was extracted as green values and converted to OD_600_ using a standard curve following the instructions provided by the supplier of the Growth Profiler (Enzyscreen B.V.). The minimum generation time and lag phase duration for each strain were calculated using the “all splines” package in R, as previously described (35). Statistical differences between the CRISPRi/a strains and the control strains were assessed using unpaired two-sample *t*-tests, and *P* values were adjusted using the Benjamini-Hochberg method (false discovery rate) (54). Statistical significance was indicated with star symbols (ns, *P* > 0.05; *, *P* ≤ 0.05; **, *P* ≤ 0.01; ***, *P* ≤ 0.001; ****, *P* ≤ 0.0001).

## RESULTS

### Binding of Pdr1 and Yap1 targets is enhanced in the presence of acetic acid

The binding of Pdr1 and Yap1 to their gene targets was investigated by ChIP-exo. In medium supplemented with 40 mM acetic acid, Pdr1 and Yap1 binding reads were increased mainly from the TSS up to 500 bp upstream (Fig. S4). In samples from cells grown without acetic acid, the binding reads ranged from −1000 to 1000 bp from the TSS. In this control setup, only a few read counts (on average 4–6) were recorded (Fig. S4).

**Fig 1:**
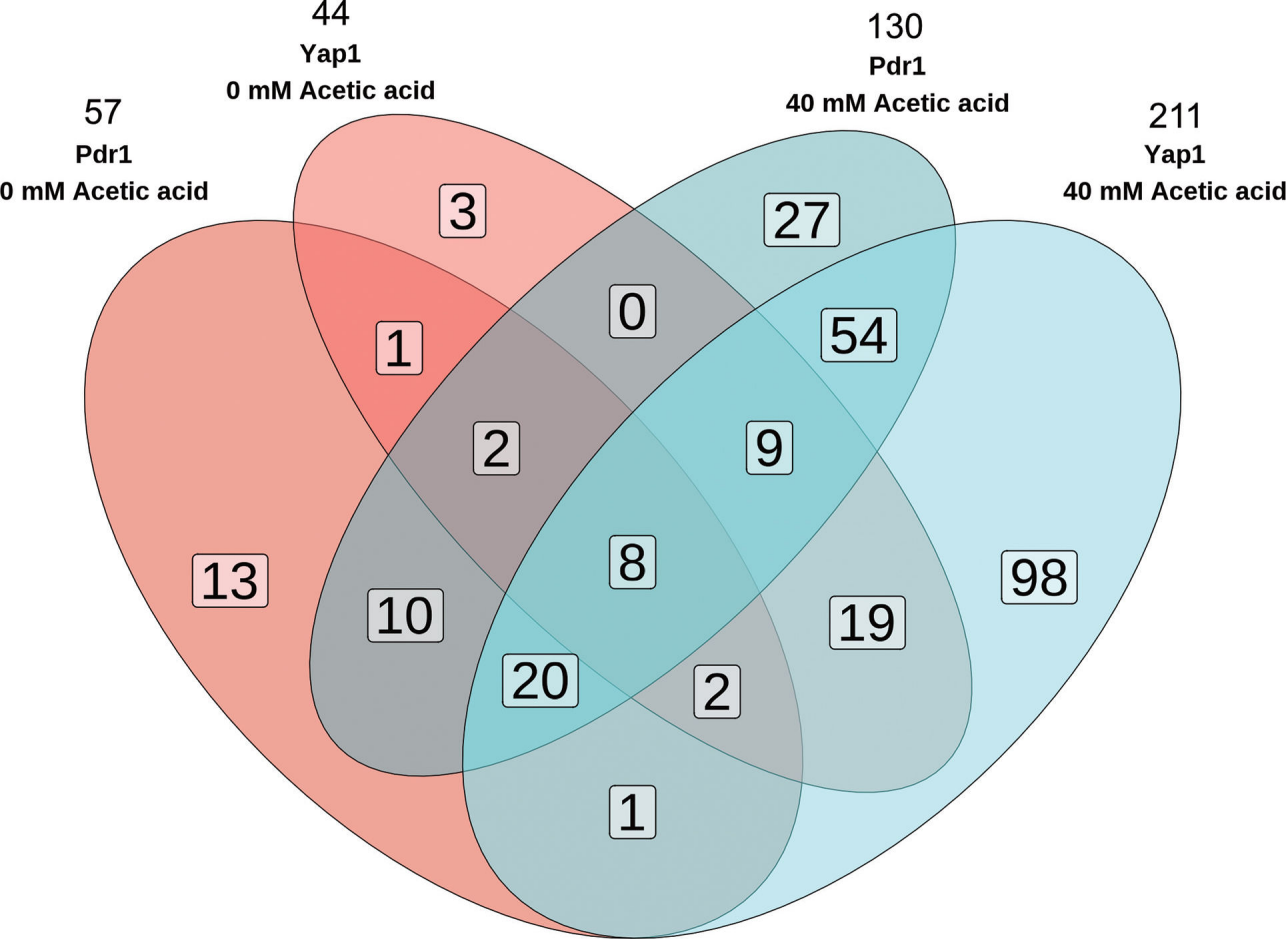
Target genes for Pdr1 and Yap1. Venn diagram representing the distribution and overlap of Pdr1 and Yap1 targeted genes at 0 (red) and 40 (blue) mM acetic acid. The total number of targeted genes is displayed above for each condition and TF.

In medium lacking acetic acid, Pdr1 targeted 57 genes, whereas in the presence of acetic acid, the number of targeted genes increased to 130, 90 of which were only targeted in the presence of acetic acid (Fig. 1; Table S1). Moreover, there were 17 genes targeted in the control condition that were not targeted in the presence of acetic acid. Yap1 targeted 44 and 211 genes in medium without and with acetic acid, respectively (Fig. 1). Out of these genes, 173 were targeted only in the presence of acetic acid (Fig. 1; Table S2), and only six genes were exclusively targeted by Yap1 in the control condition. In medium with acetic acid, 75 genes were targeted by both Pdr1 and Yap1, whereas merely 17 genes were targeted by both TFs in medium lacking acetic acid (Fig. 1; Table S3). Twelve of these genes were targeted in both media. A total of 54 genes were targeted by both Pdr1 and Yap1 only in the presence of acetic acid (Table S4).

About half, i.e., 63 of the 130 genes that Pdr1 targeted in medium with acetic acid, were known to be Pdr1 targets, and 124 of 211 genes were known Yap1 targets, according to the Yeastract database (55). GO term analysis of the genes that were targeted solely in the presence of acetic acid revealed that the significantly enriched (*P* < 0.1) genes belong to the GO process terms “Small molecule biosynthetic process,” “Carbohydrate metabolic process,” “Carboxylic acid metabolic process,” and “Small molecule metabolic process” (Table 2). A total of 48 genes targeted by Yap1, of which 16 were also targeted by Pdr1, were enriched for the GO term “Small molecule metabolic process.”

**TABLE 2:** Process categories of genes with significantly increased binding by either Pdr1 or Yap1 at 40 mM acetic acid^*a*^

GO term	Adjusted *P* value	Targeted genes of the GO term /total targeted genes	Genes of the GO term/total genes	Targeted genes	TF
Small molecule biosynthetic process	0.009	15/90	346/7166	***ILV5*** *, **ARG3**, **TDH3**, **GPP1**, **GCN4**, **OLE1**, **CPA1**, HIS1, **ERG5**, **MET6**, **LEU1**, **ERG25**, LYS20, GLY1, **PGK1***	Pdr1
Carbohydrate metabolic process	0.053	12/90	270/7166	*FKS1, KNH1, **CIT2**, **PGK1**, UTH1, **TAL1**, GAC1, **TDH3**, **GPP1**, SUN4, **TYE7**, **CDC19***	Pdr1
Carboxylic acid metabolic process	0.080	15/90	418/7166	***OLE1*** *, **CPA1**, **CDC19**, **TYE7**, HIS1, **ILV5**, **ARG3**, **TDH3**, **GCN4**, LYS20, GLY1, **PGK1**, **CIT2**, **MET6**, **LEU1***	Pdr1
Small molecule metabolic process	2.61E-7	48/173	779/7166	*TKL1, SOL3, HIS5, GND1, **GCN4**, **PGK1**, **TAL1**, **GPP1**, CPA2, **ERG25**, **ILV5**, ENO1, GPM1, YBR053C, ENO2, **ARG3**, BNA1, TPI1, **ERG5**, UGA3, HIS4, GID8, SLM5, ARG56, GLT1, ZWF1, **MET6**, **LEU1**, PDC1, URA2, DFR1, YDL124W, ADE12, YGK1, HSP31, ADH6, TRX1, **CDC19**, **TYE7**, **OLE1**, ADE2, **CIT2**, **CPA1**, **TDH3**, TDH2, ISA1, TDH1, MET17*	Yap1

^*a*^ Genes targeted by both Pdr1 and Yap1 are highlighted in bold.

### CRISPRa/i of *YAP1* and *PDR1* influenced growth in the presence of acetic acid

**Fig 2:**
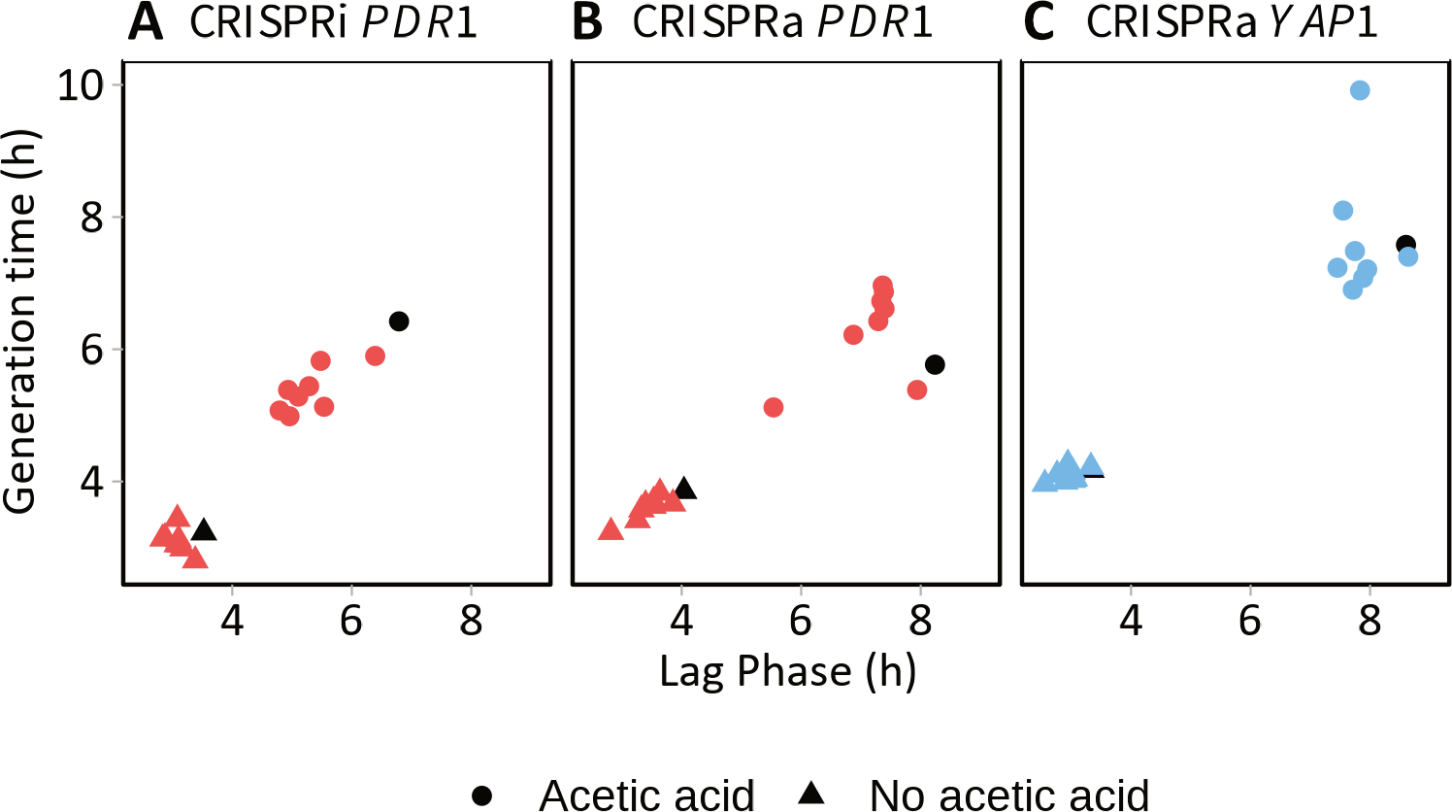
The generation time at the maximum specific growth rate plotted against the lag time of CRISPRi (**A**) and CRISPRa (**B and C**) strains at 0 (triangles) and 120 mM (dots) of acetic acid. Strains containing sgRNAs 1-8 targeting *PDR1* are marked in red, whereas the strains with sgRNAs targeting *YAP1* are marked in blue. Control strains containing no sgRNA are marked in black. Dots indicate average values of three technical replicates.

The CRISPRi/a technology was chosen to enable various levels of expression of *PDR1* and *YAP1*. The CEN.PK113-5D strain was transformed with plasmids that regulate the expression of either *PDR1* (with *dCas9-Mxi1* and *dCas9-VPR*) or *YAP1* (*dCas9-VPR*). Eight different sgRNAs for each target gene were tested, and all strains were grown at 0 and 120 mM acetic acid. In medium lacking acetic acid, all strains showed similar generation time and lag phase length (Fig. 2). In medium with acetic acid, the different sgRNAs clearly affected both the generation time and the lag phase of the strains. Notably, at 120 mM acetic acid, most CRISPRa strains had longer generation times than the CRISPRi strains (Fig. 2).

### CRISPRa targeting of *YAP1* affects acetic acid tolerance of *S. cerevisiae*

**Fig 3:**
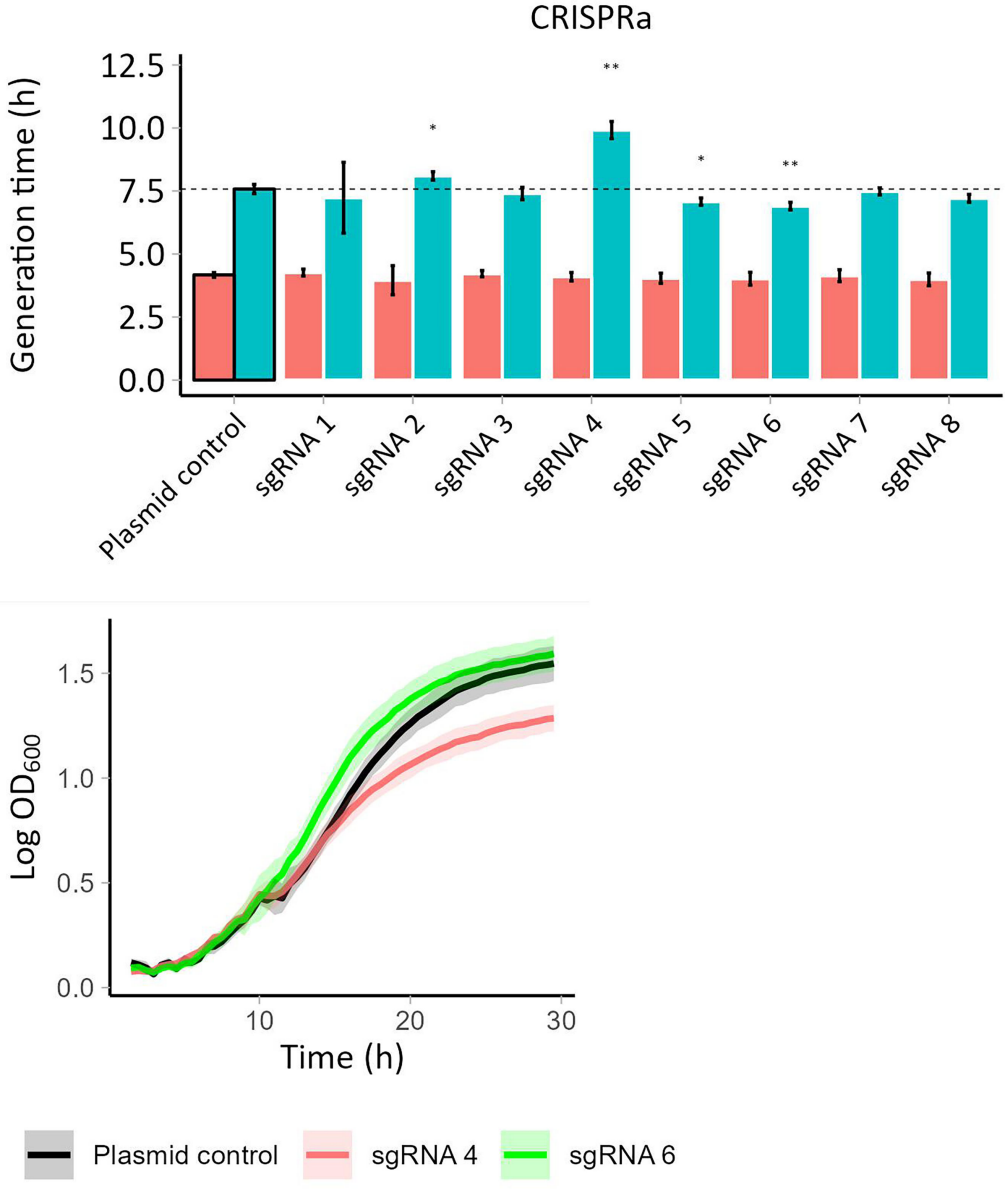
(**A**) Barplots presenting the generation time of the CRISPRa strains with sgRNAs targeting *YAP1* in medium with 0 (in red) or 120 mM (in green) acetic acid. The first bar represents the control strain with no sgRNAs. (**B**) Growth curves of YAP1a-sgRNA6 (in green) and YAP1a-sgRNA4 (in red) and the control strain (in black). Error bars show the standard deviation of three replicates. Symbols indicate statistical significance (*, *P* ≤ 0.05; **, *P* ≤ 0.01; ***, *P* ≤ 0.001).

All CRISPRa strains with sgRNAs targeting *YAP1* grew similarly in medium lacking acetic acid, except for YAP1a-sgRNA4 which had a 17% shortened lag time also in medium lacking acetic acid (Fig. S2). The CRISPRa strains expressing sgRNAs 2-8 showed a significantly shortened (8%–12%, *P* ≤ 0.05) lag phase in medium with 120 mM acetic acid (Fig. S2; Table S6). In line with this, the CRISPRa strains expressing sgRNAs 5 and 6 showed a 6 and 9% shortened generation time in medium with acetic acid, respectively (Fig. 3A; Table S7). On the contrary, two CRISPRa strains, those expressing sgRNAs 2 and 4, showed a six or 30% prolonged generation time in acetic acid-containing medium (Fig. 3A; Table S7). Notably, the YAP1a-sgRNA6 strain showed a uniform growth curve, whereas the growth of the other strains followed a diauxic growth curve, similar to the one of the control strain (Fig. 3B).

### CRISPRi targeting of *PDR1* improved growth in the presence of acetic acid

**Fig 4:**
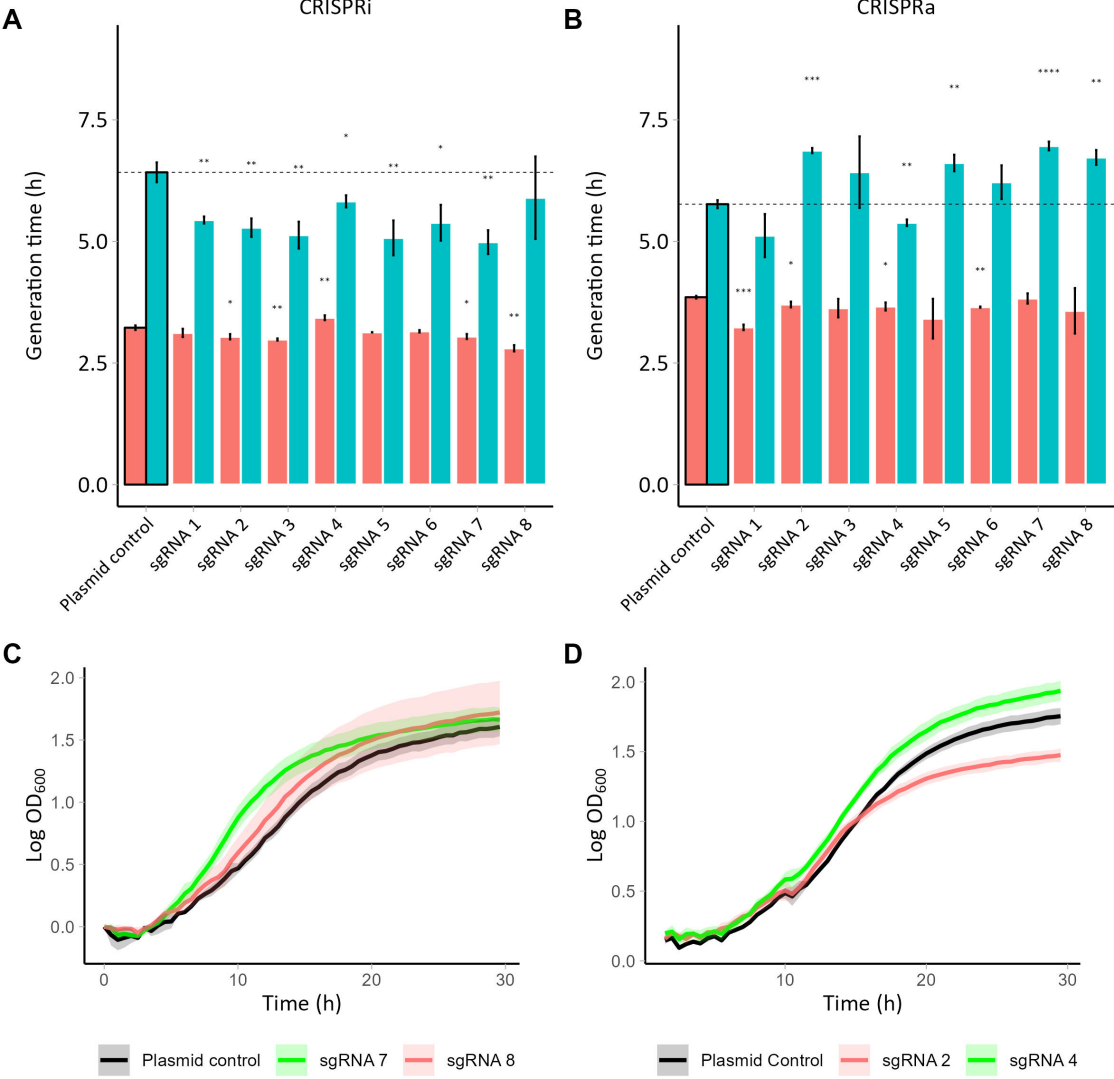
Barplots presenting the generation time of the (**A**) CRISPRi and (**B**) CRISPRa strains with sgRNAs targeting *PDR1* in medium with 0 (in red) or 120 mM (in green) acetic acid. The first bar in each graph represents the control strain with no sgRNA. (**C**) Growth curves of PDR1i-sgRNA7 (in green), PDR1i-sgRNA8 (in red), and the control strain (in black). (**D**) Growth curves of PDR1a-sgRNA2 (in green), PDR1a-sgRNA4 (in red), and the control strain (in black).

Targeting of *PDR1* with either CRISPRi or CRISPRa plasmids had a significant effect on the growth of the strains, already in medium without acetic acid (Fig. 4). Five CRISPRi strains (sgRNAs 1, 4-7) displayed significantly shorter lag phases (*P* ≤ 0.05) (Fig. S3; Table S6), whereas four CRISPRi strains targeting *PDR1* had shortened generation times (strains with sgRNAs 2, 3, 7, and 8) in medium lacking acetic acid (Fig. 4A; Table S7). Similarly, CRISPRa of *PDR1* led to a significantly shortened lag phase (by 9%–30%, *P* ≤ 0.05) of six strains (with sgRNAs 1-4 and 6-7; Fig. S3; Table S6) and a significant shortening of the generation time of four strains (with sgRNAs 1, 2, 4, and 6) in medium lacking acetic acid (*P* ≤ 0.05) (Fig. 4B; Table S7). The shortening in generation time of the CRISPRa/i strains targeting *PDR1* was relatively small compared to the control strain, 4%–16%. One strain, PDR1i-sgRNA4, had a 6% prolonged generation time in medium lacking acetic acid.

In the presence of acetic acid, all CRISPRa strains and all CRISPRi strains, except PDR1i-sgRNA8, had shortened lag phases, the most pronounced being for PDR1a-sgRNA1 that had a 32% (*P* ≤ 0.001) shorter lag phase compared to the control strain (Fig. S3). In contrast, the CRISPRa strains targeting *PDR1* tended to have longer generation times. Seven out of the eight CRISPRi strains had a significantly shortened generation time in medium with 120 mM acetic acid when compared to the control strain (by 9%–22%, *P* ≤ 0.05) (Fig. 4; Table S7). In medium supplemented with acetic acid, PDR1a-sgRNA7 was the fastest growing strain (Fig. 4B). The shortening of the lag phase ranged from 18% to 29% and was most pronounced for PDR1i-sgRNA5 (Fig. S3A; Table S6).

## DISCUSSION

In this work, we studied how two TFs, Pdr1 and Yap1, contribute to acetic acid tolerance in *S. cerevisiae*. The binding of Pdr1 and Yap1 to target genes in the presence of acetic acid was measured by ChIP-exo. The binding of these TFs was increased in the region between the TSS up to 500 bp upstream of their target genes. The highest read counts were found at approx. 300–200 bp upstream the TSS. Often, yeast promoters of >500 bp are used, and the average distance between ORFs in *S. cerevisiae* is 455 bp (56). TF binding increased in medium with acetic acid, from 24.3 reads per targeted gene in the absence of acetic acid to 54.3 reads per target in the presence of acetic acid and from 21.4 to 65.8 reads per target for Yap1 (Table S3). In line with the increased binding levels, the number of target genes also increased significantly upon exposure to acetic acid, 2.2- and 4.5-fold for Pdr1 and Yap1, respectively (Fig. 1; Table S3). This level of activation upon stress is in line with the two- to fourfold increase in gene targets that have been seen for TFs Yap4, Yap6, and Sko1 upon exposure to high salt levels (57). Under selenite stress, Yap1 has been found to bind ~300 promoters, out of which 75% contain a YRE (22). Yet, only 23% of the genes bound by Yap1 were in that study induced by selenite in a Yap1-dependent manner (22). There is some promiscuity in the YREs bound by Yap1. In our study, almost all the genes bound by Yap1 in the presence of acetic acid contained its consensus recognition element (5′-TT/GAC/GTC/AA-3′). On the contrary, only 11 of the 90 genes targeted by Pdr1 in the presence of acetic acid contained a known PDRE. Pdr1 has nonetheless been reported to occupy also degenerate PDREs (29). Workman et al. (45) studied the binding of Pdr1 upon exposure to methyl methanesulfonate (MMS), a DNA damage-causing agent. They found that Pdr1 was bound to a total of 75 genes, 64% of which were only bound in the presence of MMS. In that study, less than 50% of the Pdr1-bound genes contained a known PDRE (45).

The majority of the genes targeted by Pdr1 (67 out of 90 genes) or Yap1 (87 out of 173 genes) were not previously identified as being targeted by these TFs (as reported in the Yeastract database; Table S5). *HXT3*, encoding a low-affinity glucose transporter, was among the most targeted, previously unknown target genes of both Pdr1 and Yap1. The upregulation of *HXT3* upon acetic acid stress has been reported earlier (8), which the authors suggested could explain the sudden increase in glucose transport capacity seen in cells exposed to acetic acid (58). *CDC19*, encoding a pyruvate kinase, was also targeted by both Pdr1 and Yap1 in the presence of acetic acid. The overexpression of *CDC19* was recently shown to increase lactic acid production from a synthetic hydrolysate containing high amounts of acetic acid (59). The metabolic modeling of Choi et al. (59) that suggested *CDC19* overexpression was set to increase production from xylose and glucose. Still, the lactic acid titer in standard (YP) medium with xylose as a carbon source was not affected by the overexpression of *CDC19*. This observation together with our current study indicates that Cdc19 activity, which likely results in accelerated pyruvate accumulation, is particularly important in medium with inhibitors such as acetic acid. Carbohydrate metabolism has been reported to be strongly affected by acetic acid (60).

The GO process categories “Carbohydrate metabolic process,” “Carboxylic acid metabolic process,” and “Small molecule biosynthetic process” were significantly (*P* ≤0.1) enriched among genes targeted by Pdr1 in acetic acid-containing medium (Table 2). Notably, *TDH3*, coding for a glyceraldehyde-3-phosphate dehydrogenase involved in glycolysis and gluconeogenesis, and *GPP1*, coding for a phosphatase involved in glycerol biosynthesis, were among the genes targeted by both TFs in the presence of acetic acid. *TDH3* has previously been reported to be involved in ROS metabolism (61), and its null mutant was reported to decrease tolerance to acidic pH levels (62). *GPP1* was previously suggested to have a role in acetic acid tolerance as its expression was induced at high acetic acid levels (63). The GO term “Small molecule biosynthetic process” was enriched for genes targeted by Yap1 in this medium. Some genes belonging to this GO term encode proteins with roles in amino acid biosynthetic processes including *ARG3*, *CPA1*, *GCN4*, *ILV5*, *LEU1*, or *MET6* that were targeted by both TFs (Table S4). Altering the expression of genes involved in amino acid biosynthesis is known to affect tolerance to acetic acid (6). Deletion of *GCN4*, a leucine zipper transcription factor involved in amino acid biosynthesis (64), has previously been reported to increase the sensitivity of the cells towards acetic acid (65). Unexpectedly, another study reported that cell survivability increased for a *GCN4* null mutant treated with 140–200 mM acetic acid (60), demonstrating the complexity of genetic responses during acetic acid stress. Two genes involved in ergosterol biosynthesis were also targeted by both Pdr1 and Yap1 in the presence of acetic acid, namely, *ERG5* and *ERG25*. Many genes involved in ergosterol synthesis have been reported to be involved in tolerance to acetic acid and other lignocellulosic inhibitors (6). *ERG5* encodes a C-22 sterol desaturase, and a previous study reported that the *Δerg5* null mutant had reduced cell fitness in the presence of 0.4 (w/v) acetic acid (66). Ergosterol has been reported to play a key part in maintaining cell membrane stability in the presence of weak acids (58, 67). Pdr18, an efflux pump of the PDR network, has been proposed to mediate ergosterol incorporation and counteract acetic acid stress (67). *PDR18* has been reported to be regulated by Yap1 and Gcn4 (22), but no information on Pdr1 binding to Pdr18 has been reported (55). *PDR18* overexpression was shown to improve *S. cerevisiae*’s tolerance towards acetic acid (67), whereas its deletion increased its sensitivity (68). Notably, one of the Yap1 and Pdr1 target genes whose binding was most enhanced in acetic acid was *SNQ2* (Table S3), encoding a paralog of *PDR18*. The expression of *SNQ2* has been reported to be positively regulated by both Pdr1 and Yap1 (69, 70). Deletion of *SNQ2* did however not change the tolerance to 70 mM acetic acid (68). Thus, it seems that the roles of Pdr18 and Snq2 do not completely overlap.

Pdr1 has been described to be involved also in basal expression of PDR genes (9), which was confirmed in this study. Pdr1 is the major cell regulator for responses towards pleiotropic drugs (9, 71). Screenings of the EUROSCARF collection have revealed that deletion of *PDR1* could improve acetic acid tolerance in rich medium (11), but the *Δpdr1* strain showed no change in phenotype in minimal medium supplemented with acetic acid (72). *PDR15* (mean TF binding, 72.8) and its paralog *PDR5* (mean TF binding, 450.9), encoding plasma membrane pumps of the ATP-binding cassette (ABC) family with a major role in pleiotropic drug efflux, were among the gene targets with increased binding of Pdr1 upon acetic acid exposure (Table S3). *PDR15* has been reported to be highly induced in the presence of acetic acid stress (73), but deletion of *PDR5* did either not change sensitivity to acetic acid stress (72) or even improve acetic acid tolerance (11), further highlighting the complexity and/or condition dependence of acetic acid responses in yeast. Furthermore, acetic acid may cause different indirect effects, including oxidative stress (2).

Yap1 is known to be crucial for oxidative stress tolerance (16, 18), but the role of this TF in acetic acid tolerance has not previously been highlighted. Strains lacking *YAP1* have been reported to exhibit increased sensitivity to acetic acid, displaying reduced growth and viability under acetic acid stress (12). Moreover, overexpression of *YAP1* has been shown to lead to improved tolerance to lignocellulosic hydrolysates (74, 75) that contains acetic acid. In this study, acetic acid exposure led to a 4.5-fold increase in target binding by Yap1 (Table S3). The Yap1 targets include many genes previously reported to be involved in acetic acid tolerance, such as *CYC3* encoding a holocytochrome c synthase (76) or *TPO1* encoding a polyamine transporter (77). At 40 mM acetic acid, the promoter of *OYE2* was among the most highly abundant targets of Yap1. *OYE2* encodes an NADPH oxidoreductase involved in oxidative stress responses. Oye2 forms dimers with itself or with Oye3, a protein homolog with different ligand binding and catalytic properties. In line with the results of a previous overexpression study, Yap1-mediated induction of *OYE2* expression (78) could allow for an increase in Oye2 dimer formation and consequent increased tolerance to oxidative stress in *S. cerevisiae. OYE2* was among the most highly upregulated genes in cells adapting to acetic acid-rich lignocellulosic hydrolysates (79).

In order to determine whether changes in the transcriptional levels of *PDR1* affected cell fitness in *S. cerevisiae*, we used CRISPRi and CRISPRa technologies to modulate the TF expression and characterized the resulting strains during growth in the presence of acetic acid. Similarly, *YAP1* was targeted for VPR-mediated upregulation with eight different sgRNAs. We observed that lag phase duration of 6/8 of these strains was significantly shortened in medium with acetic acid (Fig. S2; Table S6), highlighting the critical role of *YAP1* in enhancing the ability of *S. cerevisiae* cells to tolerate high concentrations of acetic acid. With this, our study confirmed that *YAP1* upregulation has the potential to modulate strain fitness in the presence of acetic acid. As could be expected based on previous studies of Pdr1, the growth of the strains with targeting *PDR1* was highly dependent on the sgRNA used and thus presumably upon the level of expression alteration achieved. When targeting *PDR1* with dCas9-Mxi1 (Fig. 4A), most of the strains had a shortened lag phase at 120 mM acetic acid when compared to the control strain containing no sgRNA (Fig. S3A; Table S6). Similarly, the maximum generation time of 7/8 CRISPRi strains targeting *PDR1* was shortened when compared to the control strain (Fig. 4A). When targeting *PDR1* with dCas9 coupled with a VPR transcriptional activator (Fig. 4B), the lag phase of all strains was shortened (Table S6). The generation time of these strains was either prolonged (4/8 strains) or similar (3/8 strains) or even slightly shortened (1/8 strains). Thus, a reduction of the expression of *PDR1* in the cell may cause a beneficial effect on yeast tolerance to acetic acid, whereas the overexpression may be harmful. The phenotype of the strains, more profoundly seen for the CRISPRa strains targeting *PDR1*, was highly dependent on the gRNA used and parameter measured. Earlier studies have shown that effects of acetic acid on lag phase may be stronger compared to effects on generation time (24). Differences in efficiency of sgRNAs are expected due to differences in binding capacity or changing nucleosome occupancy and chromatin accessibility levels (50). It should be noted that several putative TF binding motifs were partially or totally overlapping with the locations of all the *YAP1* and *PDR1* sgRNAs (Fig. S5 and S6). This could explain the contradictory phenotypes of the different CRISPRi/a mutants (80).

### Conclusions

Our study has investigated the role of the Yap1 and Pdr1 in the cellular response towards acetic acid-induced stress in *S. cerevisiae*. In our ChIP-exo study, we observed that the number of bound target genes increased from 61 to 134 for Pdr1 and from 48 to 215 for Yap1, from which 67 new gene targets were found for Pdr1 and 87 for Yap1. GO term analysis highlighted that genes related to “Small molecule biosynthetic process” and “Small molecule metabolic process” were targeted by Pdr1 and Yap1 during acid stress. Binding to amino acid synthesis pathway genes such as *GCN4* or genes encoding ABC cell membrane transporters such as *PDR15*, *PDR5*, and *SNQ2* was enhanced for both TFs. Nevertheless, further studies on the role of the target genes in acetic acid tolerance are needed. CRISPRi/a-mediated gene expression alteration can be a fast and easy way to fine-tune the expression of crucial tolerance-related TF-encoding genes such as *YAP1* and *PDR1*. The technology can also be used to find optimal transcriptional levels of specific genes under a set condition. Our results suggest the potential of *PDR1* downregulation and *YAP1* upregulation to increase yeasts’ tolerance towards acetic acid stress.

## SUPPLEMENTAL MATERIAL

Supplemental figures aem.01824-24-s0001.docx Figures S1 to S6.

Supplemental tables aem.01824-24-s0002.xlsx Tables S1 to S9.

ASM does not own the copyrights to Supplemental Material that may be linked to, or accessed through, an article. The authors have granted ASM a non-exclusive, world-wide license to publish the Supplemental Material files. Please contact the corresponding author directly for reuse.
