# Supplementary material for: ChIP-exo and CRISPRi/a illuminate the role of Pdr1 and Yap1 in acetic acid tolerance in Saccharomyces cerevisiae
Source: Appl Environ Microbiol. 2025 Mar 4;91(4):e01824-24. doi: 10.1128/aem.01824-24 (PMC12016514; doi:10.1128/aem.01824-24)
Supplement: Supplemental figures — Figures S1 to S6. [file aem.01824-24-s0001.docx]

**Chip-exo and CRISPRi/a illuminate the role of Pdr1 and Yap1 in acetic acid tolerance in *Saccharomyces cerevisiae***

**Running title:** Chip-exo and CRISPRi/a of Pdr1 and Yap1 in yeast

**Ibai Lenitz, Christoph Börlin, Luca Torello Pianale, Darshan Balachandran, Jens Nielsen, Florian David, Verena Siewers and Yvonne Nygård**

**SUPPLEMENTARY FIGURES**


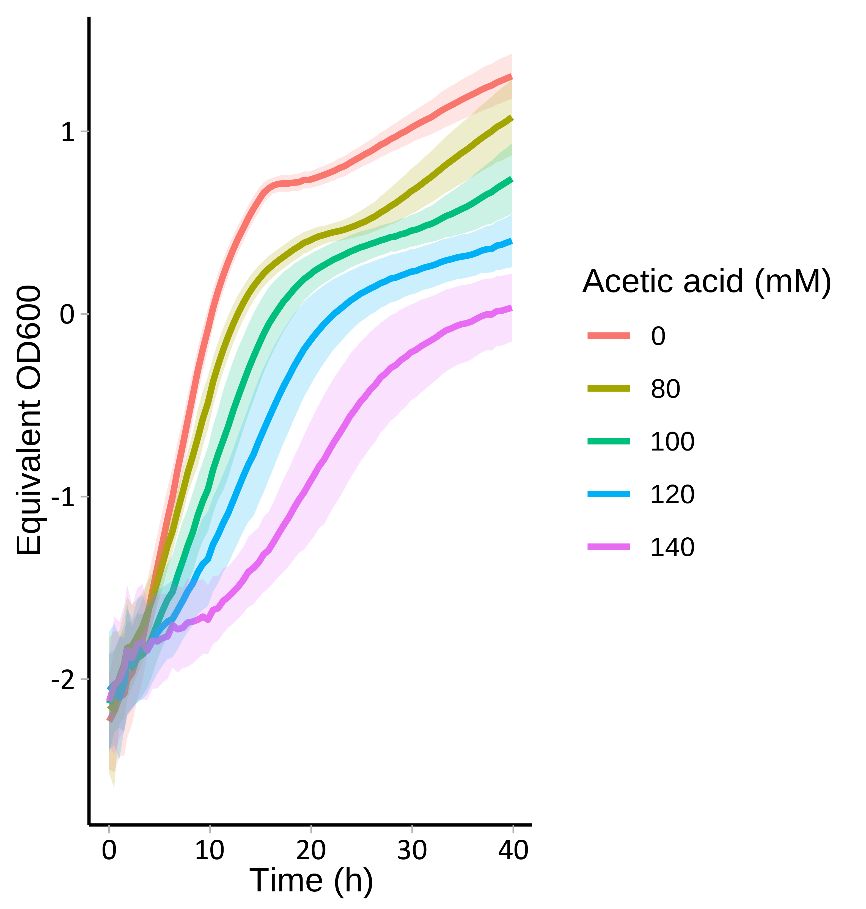


Figure S1. Graph representing the growth of the CRISPRi control strain (no sgRNA) at 0, 80, 100, 120 and 140 mM of acetic acid in YNB media. The screen was conducted to determine the acetic acid concentration to be used in the tolerance assay; 120 mM was chosen as a suitable concentration as it allowed the strains to grow, albeit displaying a delay in growth and a lower overall biomass at the stationary phase.


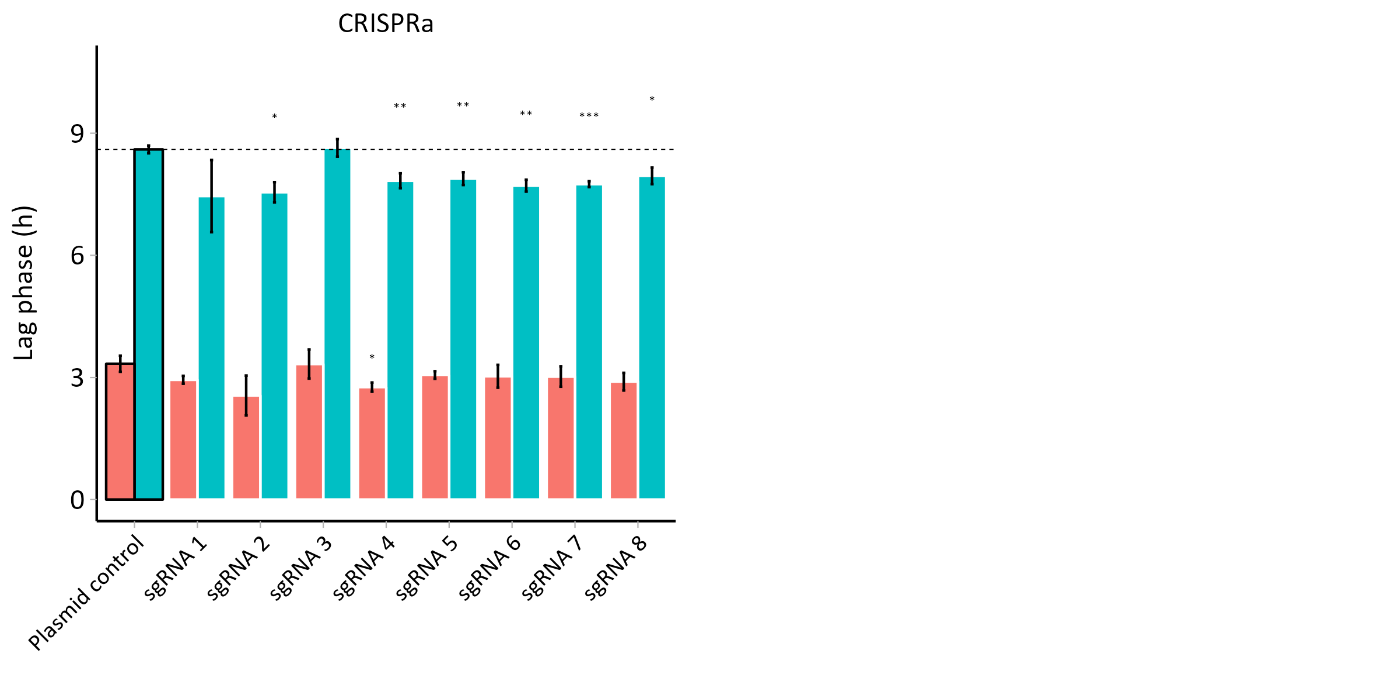


Figure S2. Barplots representing the lag phase of the CRISPRa strains with sgRNAs targeting *YAP1* in medium with 0 mM acetic acid (in red) or 120 mM (in green) acetic acid. The first bar represents the control strain with no sgRNAs. Error bars show the standard deviation of 3 replicates. Symbols indicate statistical significance (*: p ≤ 0.05; **: p ≤ 0.01; ***: p ≤ 0.001).


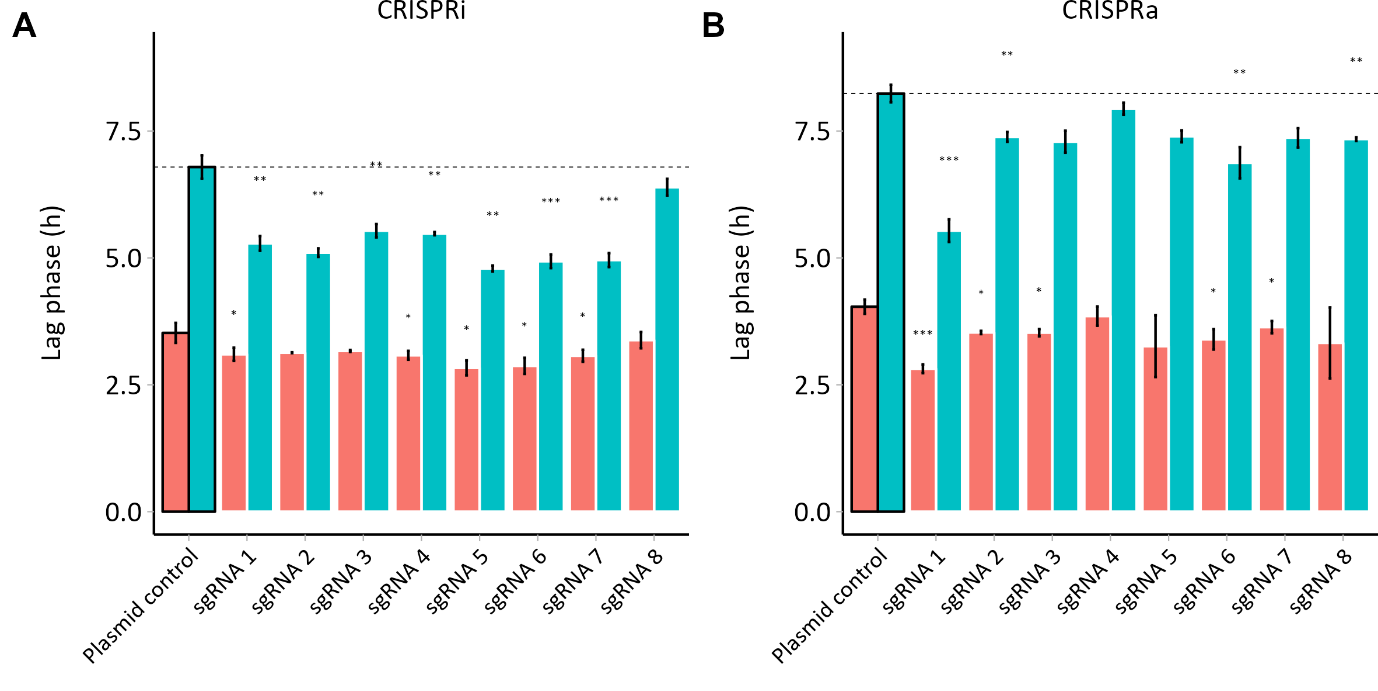


Figure S3. Barplots representing the lag phase of the CRISPRi (A) and CRISPRa (B) strains with sgRNAs targeting *PDR1*  in medium with no acetic acid (in red) or 120 mM (in green) acetic acid. The first bar of each graph represents the control strain with no sgRNAs. Error bars show the standard deviation of 3 replicates. Symbols indicate statistical significance (*: p ≤ 0.05; **: p ≤ 0.01; ***: p ≤ 0.001).


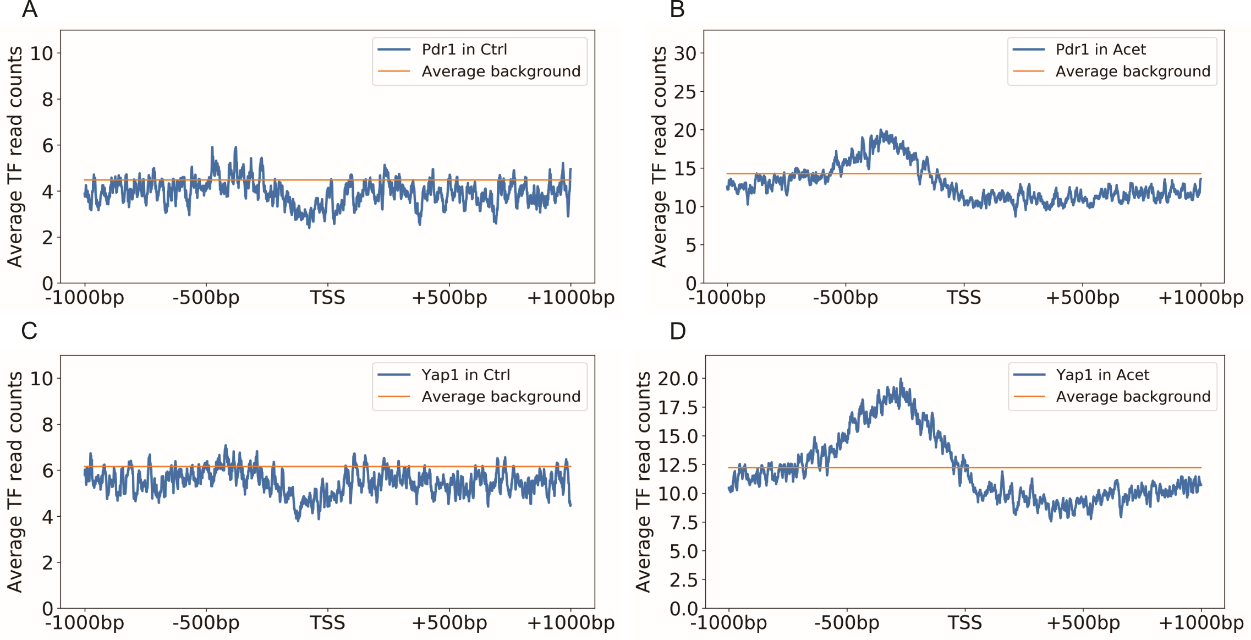


Figure S4. Average read counts for binding of Pdr1 (A,B) and Yap1 (C,D), relative to the transcription start site (TSS) of the target genes at 0 (A,C) or 40 (B,D) mM acetic acid.


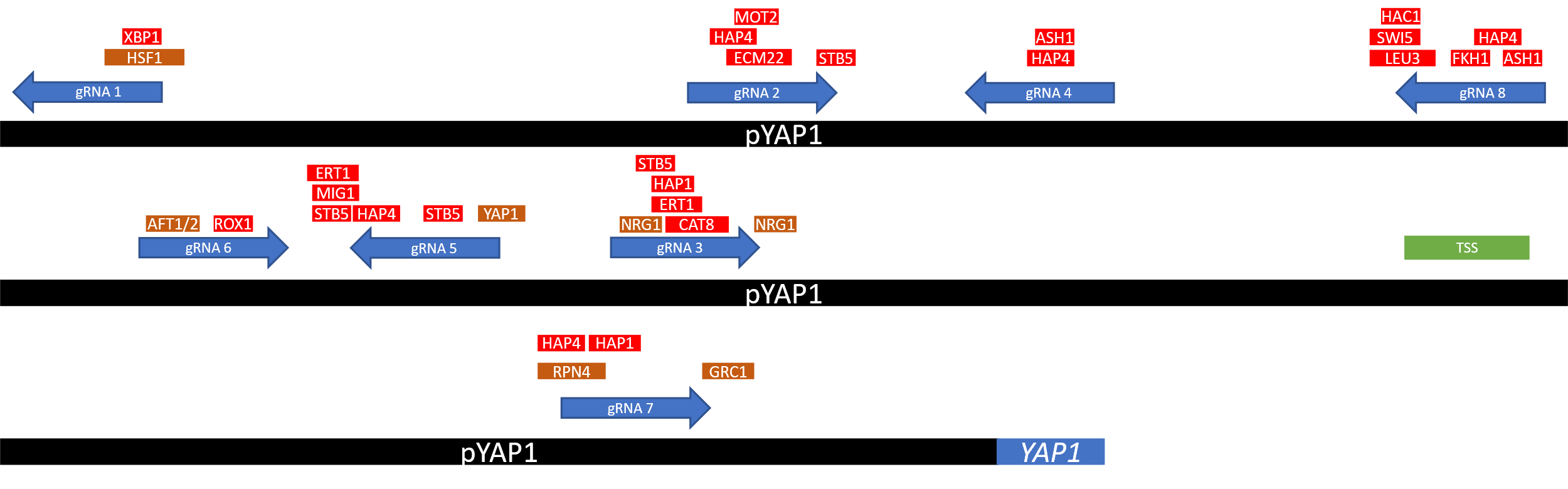


Figure S5. Promoter of *YAP1* with blue arrows indicating the gRNA target sites. Red squares indicate putative TF binding sites overlapping with the gRNA binding sites. Brown squares indicate putative binding sites of TFs previously reported to regulate *YAP1* expression. The green square shows the transcription start site (TSS).


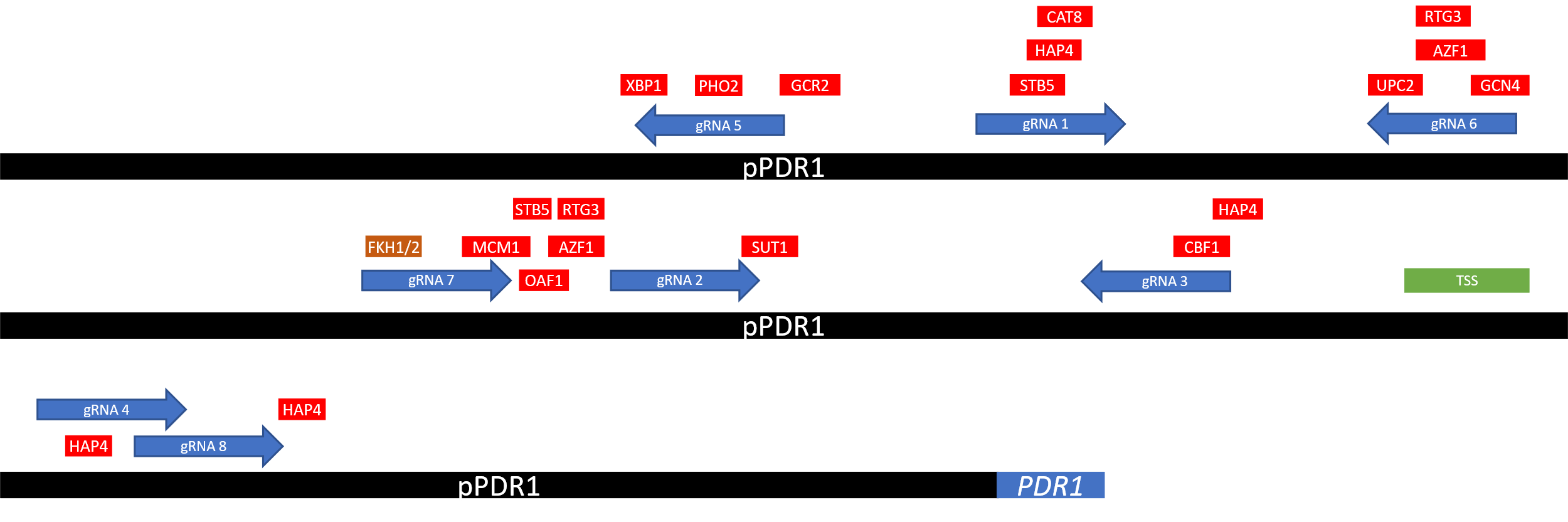


Figure S6. Promoter of *PDR1* with blue arrows indicating the gRNA target sites. Red squares indicate putative TF binding sites overlapping with gRNA binding sites. Brown squares indicate putative binding sites of TFs previously reported to regulate *PDR1* expression. The green square shows the transcription start site (TSS).
